# The influence of dexamethasone on postoperative swelling and neurosensory disturbances after orthognathic surgery: a randomized controlled clinical trial

**DOI:** 10.1186/s13005-017-0153-1

**Published:** 2017-11-07

**Authors:** W. Semper-Hogg, M. A. Fuessinger, T. W. Dirlewanger, C. P. Cornelius, M. C. Metzger

**Affiliations:** 1 Department of Oral and Craniomaxillofacial Surgery, Center for Dental Medicine, University Medical Center Freiburg, Hugstetter Straße 55 D, 79106 Freiburg, Germany; 2 Department of Oral and Maxillofacial Surgery, Ludwig-Maximilians-University Munich, Lindwurmstraße 2a D, 80337 München, Germany

**Keywords:** Dexamethasone, Swelling, Orthognathic surgery, Neurosensory disturbance, Edema, Glucocorticoids

## Abstract

**Background:**

Orthognathic surgery is associated with considerable swelling and neurosensory disturbances. Serious swelling can lead to great physical and psychological strain. A randomized, prospective, controlled clinical trial was realized in order to evaluate the effect of a preoperative intravenous dexamethasone injection of 40 mg on postoperative swelling and neurosensory disturbances after orthognathic surgery.

**Methods:**

Thirty-eight patients (27 male and 11 female) patients, all with the indication for an orthognathic surgery, were enrolled in this study (mean age: 27.63 years, range: 16–61 years) and randomly divided into two groups (study group/ control group). Both groups underwent either maxillary and/or mandibular osteotomies, resulting in three subgroups according to surgical technique (A: LeFort I osteotomy, B: bilateral sagittal split osteotomy (BSSO), C: bimaxillary osteotomy). The study group received a single preoperative intravenous injection of 40 mg dexamethasone. Facial edema was measured by 3D surface scans on the 1st, 2nd, 5th, 14th and 90th postoperative day. Furthermore, neurosensory disturbances on the 2nd, 5th, 14th and 90th postoperative day were investigated by thermal stimulation.

**Results:**

Facial edema after LeFort I osteotomy, BSSO and bimaxillary osteotomy showed a significant decrease in the study group compared to the control group (*P* = 0.048, *P* = 0.045, *P* < 0.001). The influence of dexamethasone on neurosensory disturbances was not significant for the inferior alveolar nerve (*P* = 0.746) or the infraorbital nerve (*P* = 0.465).

**Conclusions:**

Patients undergoing orthognathic surgery should receive a preoperative injection of dexamethasone in order to control and reduce edema. However, there was no influence of dexamethasone on reduction of neurosensory disturbances.

**Trial registration:**

DRKS00009033.

## Background

Several methods exist to reduce considerable swelling after orthognathic surgery. The most common method to reduce swelling is cooling [1, 2]. Glucocorticoids are also recommended to reduce postoperative edema [3]. They decrease the permeability of the capillaries [4]. Consequently there are less fluid and inflammation mediators entering the concerned tissue [5–7]. Therefore, several trials in oral and maxillofacial surgery have evaluated the use of glucocorticoids to reduce and control the postoperative side effects [5, 8–14]. All these clinical trials were not able to measure the swelling three-dimensionally due to a lack of technical capabilities, such as 3D scanning facilities. Two-dimensional measurements cannot reflect the extension of facial swelling adequately. The three-dimensional volume of swelling in its complete extension should be quantified in the present study. 3D surface scans are used more and more frequently in orthognathic and plastic surgery. Few studies in orthognathic surgery have evaluated the postoperative swelling applying this procedure by comparing cooling devices with conventional cooling. Additionally, they evaluated how body mass index, age and sex influence the swelling [2, 15].

Concerning neurosensory disturbances, one of the most frequently described complications after bilateral sagittal split osteotomy (BSSO) is an impairment of the inferior alveolar nerve (IAN) [16]. Many trials have described a large number of different testing methods to quantify these neurosensory disturbances, but comparatively few trials have evaluated a possible effect on the reduction of neurosensory disturbances of glucocorticoids [3, 17–19]. Therefore, further studies are needed [3, 20].

The aim of the present study was to investigate the swelling three-dimensionally at defined time points and to evaluate the effect of a preoperative injection of 40 mg of dexamethasone. The possible effect of glucocorticoids on reduction of neurosensory disturbances was examined.

## Methods

The study had been approved by the local ethics committee of the Albert-Ludwigs-Universität Freiburg, Germany (Protocol number: EK 4 / 14).

### Study design

**Fig. 1:**
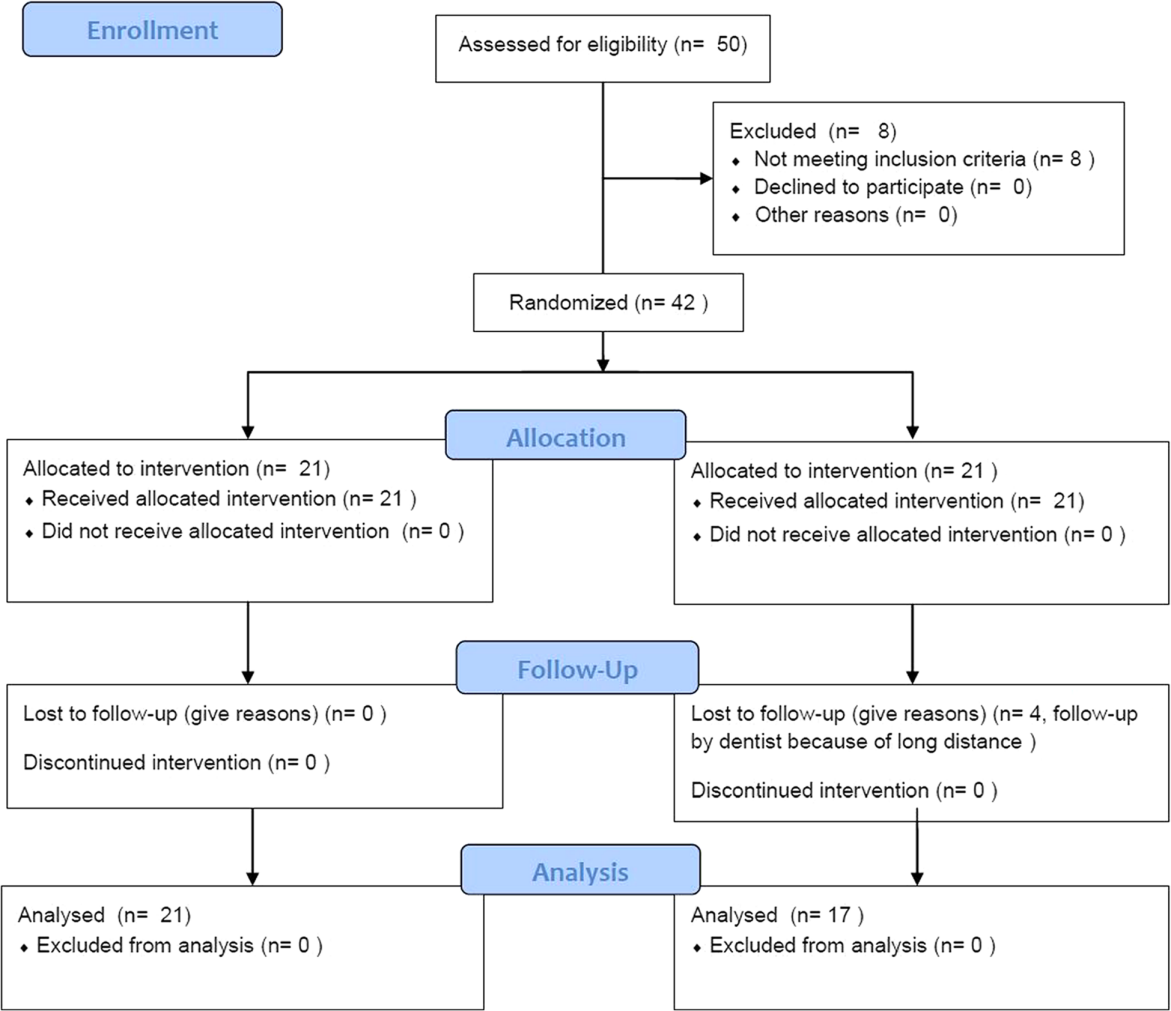
Consort Statement Flow Diagram

A randomized, prospective, controlled trial was carried out at the University of Freiburg in the Department of Oral and Maxillofacial Surgery. Thirty-eight patients (27 male and 11 female, mean age: 27.63 years, range: 16–61 years) with an indication for orthognathic surgery were enrolled in this study. Exclusion criteria were regular drug therapy, psychiatric illness, coagulopathy, diabetes mellitus, and chronical infections. The patients involved were divided into three subgroups, depending on the executed surgery (A: LeFort I osteotomy, B: BSSO, C: bimaxillary osteotomy). The patients of each group were randomly assigned to a study-group and a control-group. Baseline characteristics of the patient collective are shown in Table 1 and the Consort Statement Flow Diagram is presented in Fig. 1. The study-group received a single preoperative injection of 40 mg dexamethasone (Fortecortin Inject, Merck Serono GmbH). Surgery duration was documented in all cases. To homogenize the groups, all participants got a thorough instruction in cooling. This standardized postoperative cooling procedure was important to allow a direct comparison of the swellings. Therefore cooling was realized immediately after the operation for the time of hospitalization (for subgroups A and B 3–4 days, subgroup C 5–7 days). All participants used the same type of cooling device (Hilotherm GmbH, Argenbühl-Eisenharz, Germany) at a temperature between 17 and 19 °C for 14–16 h/day.

**Table 1:** Baseline charcteristics of the patient collective

	*n* (%)	
Ethnic group		
Caucasian	38 (100%)	
Gender		
Male	27 (71%)	
Female	11 (29%)	
Age		
< 20 years	6 (15,8%)	
20–30 years	26 (68,4%)	
30–50 years	3 (7.9%)	
> 50 years	3 (7.9%)	
Surgery	12 (31.6%)	7 study group
LeFort I		5 control group
BSSO		(2 lost to follow-up)
Bimaxillary osteotomy	16 (42.1%)	8 study group
		8 control group
	10 (26.3%)	6 study group
		4 control group
		(2 lost to follow-up)

**Fig. 2:**
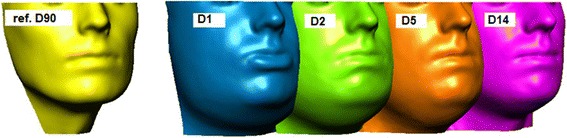
Development of swelling – D1 = first postoperative day, D2 = second postoperative day, D5 = fifth postoperative day, D14 = fourteenth postoperative day, D90 = ninetieth postoperative day (reference scan)

**Fig. 3:**
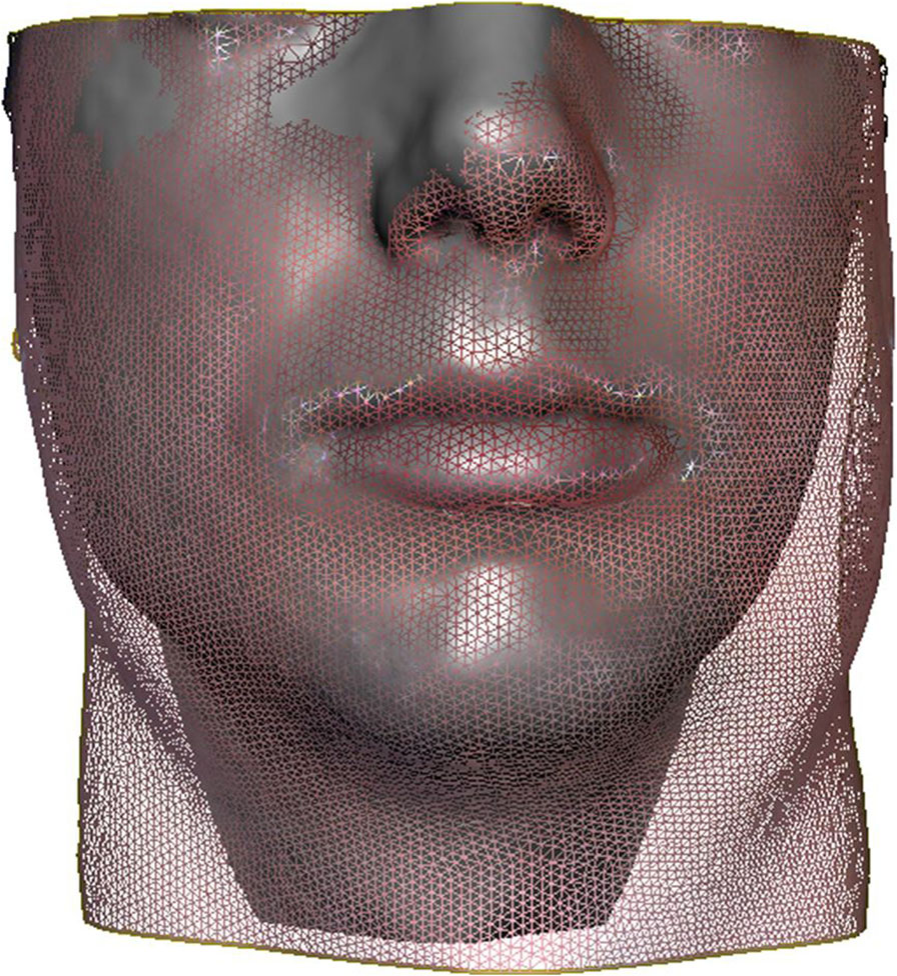
Textured face scan of D90 (grey) and polygonal mesh of D1 (red) of a female patient after bilateral sagittal split osteotomy (BSSO) demonstrating the areas with less (nose and infraorbital area, right) or maximum swelling (paramandibular area, left, and neck)

The facial swelling was determined by the three-dimensional scanning device 3dMD (Atlanta, USA) consisting of six vision cameras and a flash light. The scanning system is linked to a personal computer with the relating software 3dMD Patients (3dMD, Atlanta, USA). The result is a polygonal mesh (45.000–60.000 polygons) ready for evaluation. The amount of swelling was measured by the volume (milliliters). 3D surface scans were generated at five different points of time: D1 (first postoperative day, only for subgroups A and B, in subgroup C not possible due to physical impairment after intervention), D2 (second postoperative day), D5 (fifth postoperative day), D14 = (fourteenth postoperative day) and D90 (ninetieth postoperative day) (Figs. 2 and 3)*.* After cutting and customizing the scans by anatomical structures, scans D1-D14 were matched to the reference scan D90. The forehead and the ridge of the nose were used for surface matching because they are barely influenced by the swelling. After surface matching, the volume difference between the masks was calculated.

Neurosensory disturbances were determined by thermal stimulating using the MSA Thermal Stimulator (Somedic AB, Hörby, Sweden). It consists of a thermode and a push-button and is linked to a personal computer with the accompanying software SENSELab MSA v.6.25 (Somedic AB, Hörby, Sweden). Depending on the executed surgery, the thermode is placed on the skin above the infraorbital foramen (infraorbitale nerve / LeFort 1 osteotomy) or above the mental foramen (inferior alveolar nerve / BSSO) to evaluate heat and cold thresholds for the inferior alveolar nerve (IAN) or the infraorbital nerve (ION). Each sequence consists of ten single stimulations (five heat- / five cold stim.). The starting temperature is defined at 32 °C (max. 50 °C / min 15 °C). The heat and cold thresholds are calculated as the average of the five single stimulations. These tests were performed on the second, the fifth, the fourteenth and the ninetieth postoperative day.

### Surgical procedure

Each patient received orthodontic treatment preoperatively. The same surgeon performed all operations. Twelve patients underwent LeFort I osteotomies (7 with preoperative dexamethasone injection) and 16 a bilateral sagittal split osteotomy (8 with preoperative dexamethasone injection) as described by Obwegeser/Dal Pont [21]. Bimaxillary surgery was necessary in 10 cases (6 with preoperative dexamethasone injection).

### Statistical analysis

For a descriptive analysis mean, median and standard deviation were computed. Statistical analyses were performed by the t-test and the method of Scheffe (adjustment of *p*-values). Significance level was set on *P* < 0.05. The data were analyzed using the statistical software package STATA 13.1. (StataCorp LP, Texas, USA).

## Results

Thirty-eight patients (27 male, 11 female) were included in the study. There were no statistically significant differences between our study and control group concerning age and sex. Mean surgery duration was 97,16 (±41,29) minutes in subgroup A, 142,56 (±29,24) minutes in subgroup B, and 285 (±63,56) minutes in subgroup C.

### Postoperative swelling

The degree of swelling was measured by matching a three-dimensional mask in swollen condition (D1-D14) to a reference mask in unswollen condition (D90) and calculating its volume difference. Figs. 2 and 3 illustrates the development of swelling.

**Fig. 4:**
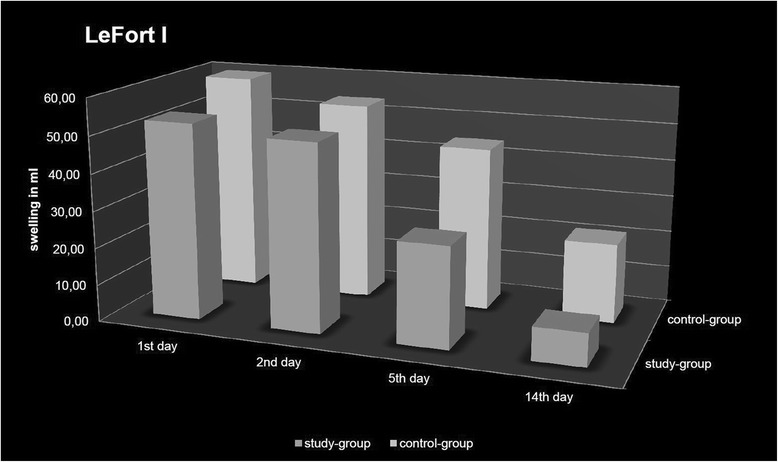
Postoperative swelling following LeFort I osteotomy (D1-D4) with and without a preoperative dexamethasone injection

The maximum swelling in the LeFort I subgroup was observed on postoperative day one with a significant difference in swelling between the study group and control group with less swelling in the study group (−11%: 53.05 ml (± 30.06) vs. 59.49 ml (±28.28), *p* = 0.048). Fourteen days postoperatively, the remaining swelling differed between the two groups (study group: 16%, 8.84 ml (±5.16) vs. control group: 36%, 21, 45 ml (±18.95) (Fig. 4)*.*

**Fig. 5:**
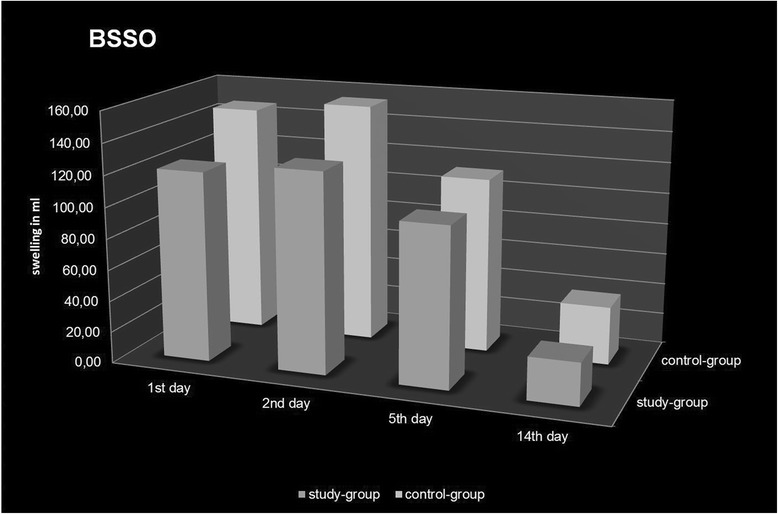
Postoperative swelling following bilateral sagittal split osteotomy (BSSO) (D1-D4) with and without a preoperative dexamethasone injection

In contrast to LeFort I osteotomies, the maximum of swelling in the BSSO patients was observed on postoperative day two. Less swelling was also observed in the study group (−18%: 127.84 ml (±21.97) vs. 153.95 ml (±53.85), *p* = 0.046). The remaining swelling 14 days postoperatively was about 25% in comparison to its initial value in both groups (study group: 21%, 27.13 ml (±18.40) vs. control group: 24%, 37.13 ml (±21.69) (Fig. 5).

**Fig. 6:**
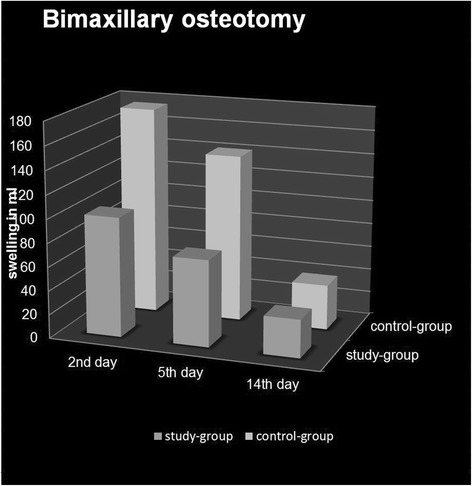
Postoperative swelling following bimaxillary osteotomy (D2-D4) with and without a preoperative dexamethasone injection

After bimaxillary osteotomies, there was 39% less swelling on postoperative day two in the study group compared to the control group (107.46 ml (±32.78) vs. 175.39 ml (±45.09), *p* < 0.001). Despite the much higher initial swelling in the control group, there were no differences in swelling 14 days postoperatively between the two groups (study group: 32%, 31.9 ml (±10, 25) vs. control group: 22%, 37.8 ml (±22.31)) (Fig. 6).

### Postoperative neurosensory disturbances

Neurosensory disturbances were quantified by thermal stimulating in 30 of the 38 patients (20 male, 10 female) of a mean age of 28.11 years.

Statistical analysis showed, that the influence of dexamethasone on the reduction of neurosensory disturbances of the IAN was not significant different between the study and control group (*P* = 0.746). Similar results were observed for the ION (*P* = 0.465).

## Discussion

Controlling and reducing postoperative edema after orthognathic surgery is important for the surgical outcome and the patient’s recovery. Even airway obstruction can occur in severe cases [22].

Three-dimensional face scans have opened the opportunity to measure the three-dimensional swelling in its complete extension without unnecessary radiation exposure [10, 23]. This procedure is described more and more frequently and has proven its precision and accuracy. The scan has to be taken with the same facial expression to ensure an adequate matching of the scans. In this study, all participants were asked to close their eyes and keep their mouths closed during image acquisition.

For the evaluation, the scan from postoperative day 90 (D = 90) was chosen as reference scan. Investigations confirm that only about 10% of the swelling can be observed at that time point and minimal facial changes occur in the following months [15, 24]. A preoperative scan would lead to wrong results as the surgery changes the patient’s facial profile. Due to the general conditions of the patients and related immobility after bimaxillary osteotomies (subgroup C), the first postoperative face scan was taken on postoperative day 2. In two of three subgroups (BSSO and bimaxillary osteotomies), the maximum swelling was observed on postoperative day 2 confirming the results of Milles et al., Troullos et al. and Lin et al. [25–27].

In contrast to a study conducted by Lin et al., no glucocorticoids were administered to the control group to investigate the medication-related effect as described by several authors [5, 8, 20]. Glucocorticoids have not yet reached full acceptance in orthognathic surgery in all medical centers [5]. Long-term glucocorticoid therapy can cause several side effects if administered with high doses and for more than 5 days [20, 28] such as Cushing-Syndrome, adrenal insufficiency, temporarily increase of blood sugar level [22, 29] psychological impairment [30] or even decrease of wound healing [31]. However, there is no clear evidence, that a single preoperative administration of glucocorticoids shows the reported side effects. Still, the dose having the highest capability in reducing swelling is unknown. The present study used 40 mg dexamethasone according to studies in traumatology. In these studies 40 mg dexamethasone is administered as antiemetic and opioid-sparing medication after surgery. Also in oncologic studies a high single dose of dexamethasone shows beneficial effects versus prednisolone. Regarding side effects, the results are somewhat contradictory. While one study showed a slight increase of complications after perioperative corticoid administration, most studies showed no side-effects of a single high-dose dexamethasone therapy [13, 32]. Due to these very positive effects, we decided in favor to the relatively high single dose amount, even if in former studies in orthognatic and oral surgery, a single shot administration of only 8-16 mg dexamethasone was used, which, however, shows only some beneficial effects concerning swelling and edema [11–14].

Regarding possible side effects of the glucocorticoids like decreased wound healing, increased infection rate hypotension, or even neurosensory disturbances, there was no difference between the control and the test group. However, the results confirm the assumption that glucocorticoids decrease postoperative edema after orthognathic surgery, which is a clear negative aspect of orthognathic surgery. The amount of swelling is inter-individually different and depends also on the surgical techniques. Differences in surgical techniques were excluded by including only cases treated by one surgeon. All in all, we were able to show that the single high dose of the glucocorticoid dexamethasone shows beneficial effects in reduction of postoperative edema, independent of the amount of swelling. Therefore, we can state that the single dose together with modern operation techniques can increase the patient’s comfort after such elective surgery.

The administration of glucocorticoids is one possibility to reduce swelling. A continuously cooling of the surgery site, shows beneficial effects, too. For this reason a standardized cooling procedure was performed in all patients to ensure comparable conditions. As described by several authors, the patients of the study group showed less swelling in the initial postoperative phase than the patients of the control group [33–36]. Comparing three different cortisone drugs (methylprednisolone, betamethasone and dexamethasone), dexamethasone seems most suitable because of its high anti-inflammatory activity, no mineralocorticoid activity and its long biologic half-life of 36–48 h [5, 8, 9]. Intravenous injection is recommended preoperatively [20]. However, the ideal timing is uncertain [20]. The conversion of dexamethasone which is delivered as a prodrug takes 10 min [37]. To reach clinical effectiveness it takes about one to two additional hours [38, 39]. Therefore, an early injection 12 h preoperatively as described by Schaberg et al. does not seem to be necessary [9]. Additional postoperative injections showed no further decrease of edema [5, 8]. The different protocols as well as the different measuring methods do not allow a direct comparison of the results. Further trials with comparable and objective measurements of swelling are needed to achieve a consensus about the ideal dose and the injection time.

The present study did not approve a significant influence of dexamethasone on reduction of neurosensory disturbances. To detect possible effects, thermal stimulation was used. Many approaches of examining neurosensory disturbances have been made. They vary from analysis with visual analogue scales to the investigation of mechanical thresholds by Semmes aesthesiometer (measuring device for tactile sensitivity), thermal stimulation, light touch, static 2-point touch and pin-prick discrimination [6, 7, 18]. The variety of testing methods makes it difficult to compare the results. Al-Bishri et al. described less neurosensory disturbances after the administration of glucocorticoids. However, this trial was only based on the patients’ personal evaluation of neurosensory dysfunctions [18]. Seo et al. achieved significant less neurosensory disturbances with a glucocorticoid therapy over 2 weeks (30 mg prednisolone for 7 days, 15 mg for 4 days and 5 mg for 3 days), if started 3 weeks postoperative. A local intraoperative application of dexamethasone on the nerve shows no clinical effect as well as a single preoperative application [5, 19]. The results were gained by using thermal stimulation as a simple and precise method to measure neurosensory disturbances. Limitations based on interindividual differences must be taken into account, if judging the results. Lacking of precise and easy to handle instruments, the thermal stimulation test seems to be appropriate to compare the study and the control group. Ongoing research must increase the evidence of glucocorticoids for reduction of neurosensory disturbances. Furthermore, postoperative nausea and vomiting (PONV) are common complications after orthognathic surgery and occur in 40% of cases during the first 24 h [40]. A large systematic literature research has revealed that a single dose of dexamethasone is additionally effective in decreasing the risk of PONV [41].

## Conclusion

The present study demonstrates that dexamethasone significantly reduces postoperative swelling. In patients with bimaxillary osteotomies, the greatest reduction in swelling was achieved. A promotion of nerve healing or less neurosensory disturbances after a single injection of glucocorticoids could not be observed. Further studies are needed to evaluate the ideal dose and timing of the administration of glucocorticoids to reach the maximum benefit from dexamethasone.

## Abbreviations

BSSO: Bilateral sagittal split osteotomy
IAN: Inferior alveolar nerve
ION: Infraorbital nerve
PONV: Postoperative nausea and vomiting
